# The role of radiotherapy in the management of POEMS syndrome

**DOI:** 10.1186/s13014-014-0265-8

**Published:** 2014-11-28

**Authors:** Yang-Gun Suh, Young-Suk Kim, Chang-Ok Suh, Yu Ri Kim, June-Won Cheong, Jin Seok Kim, Jaeho Cho

**Affiliations:** Department of Radiation Oncology, Yonsei University College of Medicine, 50-1 Yonsei-ro, Seodaemun-gu, Seoul, 120-752 South Korea; Division of Hematology, Department of Internal Medicine, Yonsei University College of Medicine, Seoul, South Korea

**Keywords:** Chemotherapy, Osteosclerotic myeloma, Castleman’s disease, Monoclonal gammopathy, Polyneuropathy

## Abstract

**Background:**

POEMS syndrome is a paraneoplastic syndrome caused by an underlying plasma cell proliferative disease. In this study, we examined the treatment outcomes and role of radiotherapy in the management of POEMS syndrome.

**Methods:**

In total, 33 patients diagnosed with POEMS syndrome were analyzed. These patients presented with osteosclerotic myeloma (OSM, n = 13), Castleman’s disease (CD, n = 4), OSM with CD (n = 10), and vascular endothelial growth factor elevation without gross lesions (VEGFe, n = 6), respectively. The patients were treated by radiotherapy alone (n = 4), chemotherapy alone (n = 16), or a combination thereof (n = 9).

**Results:**

The clinical response rates of radiotherapy, chemotherapy, and radiotherapy plus chemotherapy were 75%, 69%, and 89%, respectively. In addition, the hematologic response rates were 50%, 69%, and 71%, respectively. Among the six patients with limited multiple lesions who underwent radiotherapy, the clinical symptoms were improved in five patients after radiotherapy. The median progression-free survival (PFS) was 51 months, and the median overall survival (OS) was 65 months. In univariate analysis, the administration of chemotherapy was significantly associated with better PFS (*p* = 0.007) and OS (*p* = 0.020). In contrast, underlying VEGFe was a significant factor worsening PFS (*p* = 0.035) and OS (*p* = 0.008).

**Conclusions:**

Radiotherapy produces a reliable clinical response and is effective in improving POEMS-associated symptoms that are refractory to chemotherapy in selected patients with clustered or limited multiple lesions that can be covered by single radiation field.

## Background

The term POEMS is derived from the primary features of the disease: polyneuropathy, organomegaly, endocrinopathy, M protein, and skin changes [1]. POEMS syndrome is a paraneoplastic syndrome caused by an underlying plasma cell disorders such as osteosclerotic myeloma (OSM) or Castleman’s disease (CD) [2,3]. Polyneuropathy is a typical symptom of this syndrome, and can be fatal if it is not improved with treatments [4-6]. Other important clinical features, which are not included in the POEMS acronym, include papilledema, thrombocytosis/erythrocytosis, elevated vascular endothelial growth factor (VEGF) levels, and extravascular volume overload such as peripheral edema, ascites, pleural effusion, and pericardial effusion [6-9]. Since there is no single diagnostic test for the diagnosis of POEMS syndrome, the diagnosis is typically based on the presence of polyneuropathy associated with monoclonal plasma cell disorders presenting with the distinct symptoms described above. Currently, the Mayo Clinic POEMS diagnostic criteria are generally accepted and widely used [3,6,10].

Previous studies have shown that radiotherapy and chemotherapy are effective treatments for solitary and multiple lesions, respectively [10-13]. Sometimes, surgery can be an option for patients with cranial vault plasmacytoma [14]. However, the role of radiotherapy in patients with multiple lesions remains unclear, and the treatment outcomes vary according to the underlying plasma cell disorders [15].

In the current study, we analyzed the effects of various treatments, including radiotherapy, chemotherapy, or combined treatment on the symptoms and disease control in patients with POEMS syndrome.

## Methods

### Patients

We retrospectively reviewed the medical records of 33 patients who met diagnostic criteria for POEMS syndrome, as proposed by the Mayo clinic [7] and treated at the Yonsei University Health System in Seoul, Korea, in the period between March of 2000 and February of 2013. Patients were diagnosed with POEMS syndrome if they met the two mandatory major criteria (polyneuropathy and monoclonal plasma cell-proliferative disorder), at least one of the other major criteria (CD, sclerotic bone lesion, and VEGF elevation), and at least one of the minor criteria (organomegaly, extravascular volume overload, endocrinopathy, skin changes, papilledema, and thrombocytosis/polycythemia). Plasma cell-proliferative disorders were classified into four groups: OSM, CD, OSM with CD, and VEGF elevation without gross lesions. All patients underwent pretreatment evaluation including a complete blood cell count (CBC), blood chemistry analysis, serum and urine electrophoresis and immunofixation to detect M-protein, whole body bone scan (WBBS), positron emission tomography/computed tomography, and bone marrow biopsies from the bilateral iliac bones; in patients treated after 2007, the serum VEGF levels were measured. To identify the gross aggregation of plasma cells in the bone marrow, immunohistochemistry for syndecan-1 (CD138) was performed.

The study was approved by the Institutional Review Board of the Yonsei University Health System.

### Follow-up

The treatment responses were evaluated on the basis of several outcomes, including subjective symptom improvements reported by the patients or treating physicians, and physical examination findings. Furthermore, a nerve conduction velocity test, serum immunoglobulin (Ig)A/G/M, lambda/kappa chain, and imaging studies were also performed to evaluate the treatment responses. Clinical responses were evaluated by assessing improvements in symptoms associated with POEMS syndrome, such as polyneuropathy, skin changes, pulmonary edema, and extravascular volume overload. Clinical response was categorized as improved, mixed, stable, or progressed according to previous study [12], and only the patients who experienced clinical symptom improvement were considered as responder. Hematologic responses were defined using the criteria from a previous study [11]: complete hematologic response (CR_H_), no abnormal plasma cell aggregation in the bone marrow and negative immunofixation in the serum and urine; very good partial response (VGPR_H_), a 90% reduction in the M-protein levels or positive immunofixation, as long as M-protein level was at least 0.5 g/dL at baseline; and partial hematologic response (PR_H_), a 50% reduction in serum M-protein levels. Other cases were defined as no hematologic response (NR_H_). Follow-up monitoring included CBC, blood chemistry analyses, serum and urine assays to detect M-protein, and imaging studies of the treated areas. Progression was defined as any event as follows; increase in the M-component in the serum or urine, aggravation of symptoms associated with POEMS syndromes, or progression of disease observed upon imaging studies.

### Statistical analysis

Progression-free survival (PFS) and overall survival (OS) were quantified from the date of diagnosis to the event of interest. Statistical analyses were conducted using SPSS version 20 (SPSS Inc., Chicago, IL). A *p* value <0.05 was considered statistically significant. Differences in clinical features and response rates between patient groups were analyzed using the Pearson's Chi-squared test. Patient survival was evaluated using Kaplan-Meier survival curves, and the log-rank test was used to compare survival rates between groups. Prognostic factors for survival were analyzed by univariate analyses using the log-rank test.

## Results

### Patient characteristics

The clinical and laboratory characteristics of the patients at the time of diagnosis are summarized in Table 1. All patients presented with a sensorimotor polyneuropathy in nerve conduction test. Sixteen (48%) patients had a poor performance status of Eastern Cooperative Oncology Group performance score (3), owing to the presence of peripheral polyneuropathy. All patients presented with more than four features included in the Mayo Clinic diagnostic criteria, and 42% of patients presented with more than seven features. Thirty (91%), one (3%), and two (6%) patients presented with lambda, kappa, and both light chains, respectively. The median frequency of plasma cells in the bone marrow was 3% (range, 0-18%).

**Table 1:** **Patient clinicodemographic characteristics and laboratory findings**

**Characteristic**	**All (n = 33)**	**RT (** ***n*** **= 13)**	**Non-RT (** ***n*** **= 20)**	
	**No. of patients (%)**	**No. of patients (%)**	**No. of patients (%)**	***p*** **value** ^*^
Median age, year (range)	45 (25–68)	51 (25–68)	45 (28–63)	0.592
Gender				
Male	24 (73)	12 (92)	12 (60)	0.056
Female	9 (27)	1 (8)	8 (40)	
ECOG performance status				
1	6 (18)	2 (15)	4 (20)	0.451
2	11 (33)	6 (46)	5 (25)	
3	16 (48)	5 (39)	11 (55)	
Number of POEMS features				
≤7 features	19 (58)	7 (54)	12 (60)	1.000
>7 features	14 (42)	6 (46)	8 (40)	
Type of M-protein				
IgG	17 (52)	8 (62)	9 (45)	0.305
IgA	13 (39)	5 (38)	8 (40)	
IgG and IgA	3 (9)	0 (0)	3 (15)	
Type of plasma cell-proliferative disorder				
OSM	13 (39)	8 (62)	5 (25)	0.067
CD	4 (12)	0 (0)	4 (20)	0.136
OSM + CD	10 (30)	5 (38)	5 (25)	0.461
VEGF elevation without gross lesion	6 (18)	0 (0)	6 (30)	0.060
Plasma cell component in bone marrow				
≤5%	20 (61)	8 (62)	12 (60)	1.000
>5%	13 (39)	5 (38)	8 (40)	
Abnormal clonal plasma cells in bone marrow				
Absent	28 (85)	13 (100)	15 (75)	0.131
Present	5 (15)	0 (0)	5 (25)	
Laboratory findings				
ESR >20 mm/h	16 (48)	5 (38)	11 (55)	1.000
Hemoglobin <11 g/dL	2 (6)	0 (0)	2 (10)	0.508
Hemoglobin >16 g/dL	5 (15)	4 (31)	1 (5)	0.066
Platelets >450 x 10^3^/μL	11 (33)	2 (15)	9 (45)	0.128
Creatinine clearance <60 mL/min	5 (15)	0 (0)	5 (25)	0.131

RT, radiotherapy; ECOG, Eastern Cooperative Oncology Group; Ig, immunoglobulin; OSM, osteosclerotic myeloma; CD, Castleman’s disease; VEGF, vascular endothelial growth factor; ESR, erythrocyte sedimentation rate.

^*^The *p* value was calculated by the Pearson’s Chi-squared test between RT and non-RT.

The features of POEMS syndrome according to the administration of radiotherapy are summarized in Table 2. Peripheral polyneuropathy, one of the major diagnostic criteria, was observed in all patients. All patients presented with polyneuropathy in the lower extremities, and 10 patients also had polyneuropathy in the upper extremities. For the 10 patients with polyneuropathy in both the upper and lower extremities, the symptoms had presented in the lower extremities first. Elevated protein levels in the cerebrospinal fluid were observed in 18 patients among 20 patients with available data (90%). The frequencies of extravascular volume overload including ascites (*p* = 0.009), and pleural effusion (*p* = 0.004) and pericardial effusion (*p* = 0.027) were significantly lower in patients who underwent radiotherapy. The frequency of patients presenting bone lesions was significantly higher in patients treated with radiotherapy compared to all other patients (100% versus 50%, *p* = 0.008).

**Table 2:** **POEMS features of the study patients**

**Characteristic**	**All (n = 33)**	**RT (** ***n*** **= 13)**	**Non-RT (** ***n*** **= 20)**	***p*** **value** *
	**No. of patients (%)**	**No. of patients (%)**	**No. of patients (%)**	
Polyneuropathy				
Peripheral neuropathy	33 (100)	13 (100)	20 (100)	N/A
CSF protein >50 mg/dL	18/20 (90)	5/5 (100)	13/15 (87)	1.00
Organomegaly				
Hepatomegaly	16 (48)	6 (46)	10 (50)	1.000
Splenomegaly	18 (55)	7 (54)	11 (55)	0.614
Lymphadenopathy	15 (45)	5 (38)	10 (50)	0.722
Endocrinopathy				
Diabetes mellitus	6 (18)	3 (23)	3 (15)	0.659
Hypothyroidism	15 (45)	3 (23)	12 (60)	0.072
Gonadal axis abnormality	10 (30)	3 (23)	7 (35)	0.701
Adrenal axis abnormality	8 (24)	5 (38)	3 (15)	0.213
Hyperparathyroidism	0 (0)	0 (0)	0 (0)	N/A
Skin changes				
Hyper-pigmentation	25 (76)	11 (85)	14 (70)	0.431
Acrocyanosis and plethora	1 (3)	0 (0)	1 (5)	1.000
Hemangioma/telangiectasia	0 (0)	0 (0)	0 (0)	N/A
Hypertrichosis	16 (48)	6 (46)	10 (50)	1.000
Extravascular volume overload				
Peripheral edema	19 (58)	6 (46)	13 (65)	0.472
Ascites	12 (36)	1 (8)	11 (55)	0.009
Pleural effusion	13 (39)	1 (8)	12 (60)	0.004
Pericardial effusion	7 (21)	0 (0)	7 (35)	0.027
Types of bone lesion				
Osteolytic	4 (12)	2 (15)	2 (10)	0.488
Osteosclerotic	14 (42)	7 (54)	7 (35)	
Mixed	5 (15)	4 (31)	1 (5)	
No. of bone lesion				
None	10 (30)	0 (0)	10 (50)	0.008
Solitary	11 (33)	7 (54)	4 (20)	
Multiple	12 (36)	6 (46)	6 (30)	

RT, radiotherapy; CSF, cerebrospinal fluid; N/A, not applicable.

^*^The *p* value was calculated by the Pearson’s Chi-squared test between RT and non-RT.

Radiotherapy was administered to 13 patients (39%) presenting with OSM with or without CD, and non of these patients presented with abnormal clonal plasma cells in the bone marrow. Almost patients (n = 10) treated with radiation doses of 40 Gy or more. Twenty-five patients (76%) were treated with chemotherapy. Four patients did not undergo definitive radiotherapy or chemotherapy due to low performance status or patients’ refusal; three of these patients were instead treated with prednisolone or dexamethasone alone, while one patient received prednisolone and intravenous immunoglobulin. Nine patients received both radiotherapy and chemotherapy. Detailed treatment features are summarized in Table 3.

**Table 3:** **Treatments**

**Variable**	**No. of case (%)**	**Median**	**Range**
Radiotherapy	13 (100)		
Irradiated site			
Spine	6 (46)	N/A	N/A
Pelvic bone	5 (38)	N/A	N/A
Lymph nodes	2 (15)	N/A	N/A
No. of irradiated bone lesions			
Single	7 (54)	1	1
Multiple	6 (46)	4	2-6
Total dose, Gy	13 (100)	45	30-50
Fraction size, Gy	13 (100)	2	1.8-3
Chemotherapy	25 (100)		
Melphalan and prednisolone	4 (16)	N/A	N/A
Vincristine, doxorubicin, and dexamethasone	2 (8)	N/A	N/A
High-dose chemotherapy with APBSCT	15 (60)	N/A	N/A
Others	4 (16)	N/A	N/A

N/A, non applicable; APBSCT, autologous peripheral blood stem cell transplantation.

### Treatment responses

**Figure 1:**
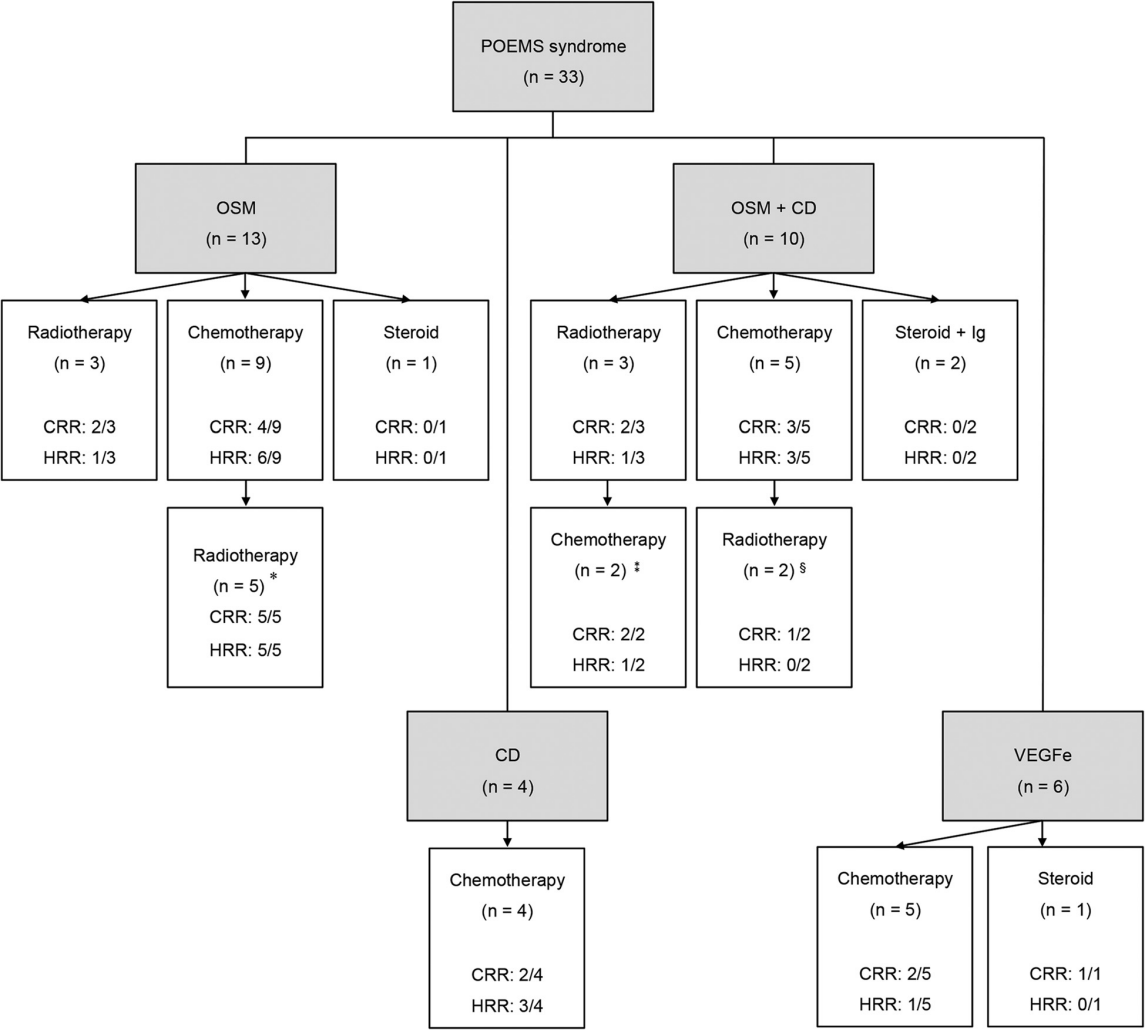
**Clinical and hematologic responses of initial and salvage treatments according to underlying disease types of POEMS syndrome.** OSM, osteosclerotic myeloma; CD, Castleman’s disease; VEGFe, vascular endothelial growth factor elevation without gross lesion; Ig, immunoglobulin; CRR, clinical response rate; HRR, hematologic response rate. *In these patients, the clinical symptoms were not improved by chemotherapy. ^⁑^In these patients, the serum M-protein persisted after chemotherapy. ^§^In these patients, the clinical symptoms and hematologic status were not improved by chemotherapy.

The various treatments and the responses according to the underlying disease are summarized in Figure 1. Among the 23 patients presenting with OSM (with or without CD), six and 14 patients were initially treated with radiotherapy and chemotherapy, respectively. The proportions of patients showing improved clinical symptoms after the initial treatment were 67% and 50% in the radiotherapy and chemotherapy groups, respectively. Out of the 14 patients who initially received chemotherapy, seven patients subsequently received radiotherapy due to poor clinical responses to chemotherapy alone. Among these patients, three patients presented with a single bone lesion, whereas four patients had multiple bone lesions more than three. After salvage radiotherapy, six of these seven patients showed clinical response.

Six patients with multiple lesions underwent radiotherapy due to poor performance status deemed inadequate for chemotherapy (n = 2), and poor clinical symptom response after chemotherapy (n = 4). Out of these six patients, the clinical symptoms were improved in five patients after radiotherapy, although the serum M-protein levels were reduced in only one patient by radiotherapy. The 10 patients with CD or VEGF elevation without gross lesions were treated with chemotherapy (n = 9) or prednisolone and immunoglobulin (n = 1).

Overall, 23 patients (70%) showed improved clinical symptoms after treatments. The clinical response rates for the patients treated with radiotherapy alone, chemotherapy alone, and chemoradiotherapy were 75%, 69%, and 89%, respectively, and these were not statistically different according to the treatments (*p* = 0.528). In addition, two (50%), 11 (69%), and six patients (67%) showed hematologic response after radiotherapy alone, chemotherapy alone, and chemoradiotherapy, respectively. No significant differences were observed between the treatment groups in hematologic response rate (*p* = 0.777). OSM patients with or without CD showed better clinical and hematologic responses compared with patients with CD only or VEGF elevation without gross lesions (Table 4).

**Table 4:** **Treatment responses according to treatment modality and type of plasma cell-proliferative disorder**

**Feature**	**No. of Pts,**	**Clinical response**				**Hematologic response**			
		**No. of patients (%)**				**No. of patients (%)**			
		**Improved**	**Mixed**	**Stable**	**Progression**	**CR**_**H**_	**VGPR**_**H**_	**PR**_**H**_	**NR**_**H**_
RT	4	3 (75)	0 (0)	1 (25)	0 (0)	1 (25)	1 (25)	0 (0)	2 (50)
CHT	16	11 (69)	1 (6)	2 (13)	2 (13)	5 (31)	2 (13)	4 (25)	5 (31)
RT + CHT	9	8 (89)	0 (0)	0 (0)	1 (11)	4 (44)	1 (11)	1 (11)	3 (33)
Steroid or Ig	4	1 (25)	1 (25)	1 (25)	1 (25)	0 (0)	0 (0)	0 (0)	4 (100)
OSM	13	11 (84)	0 (0)	1 (8)	1 (8)	6 (46)	1 (8)	3 (23)	3 (23)
CD	4	2 (50)	0 (0)	1 (25)	1 (25)	0 (0)	1 (25)	2 (50)	1 (25)
OSM + CD	10	7 (70)	1 (10)	1 (10)	1 (10)	3 (30)	2 (20)	0 (0)	5 (50)
VEGFe	6	3 (50)	1 (17)	1 (17)	1 (17)	1 (17)	0 (0)	0 (0)	5 (83)

Pts, patients; CR, complete response; VGPR, very good partial response; PR, partial response; NR, no response; RT, radiotherapy; CHT, chemotherapy; Ig, immunoglobulin; OSM, osteosclerotic myeloma; CD, Castleman’s disease; VEGFe, vascular endothelial growth factor elevation without growth lesion.

### Survivals and prognostic factors

**Figure 2:**
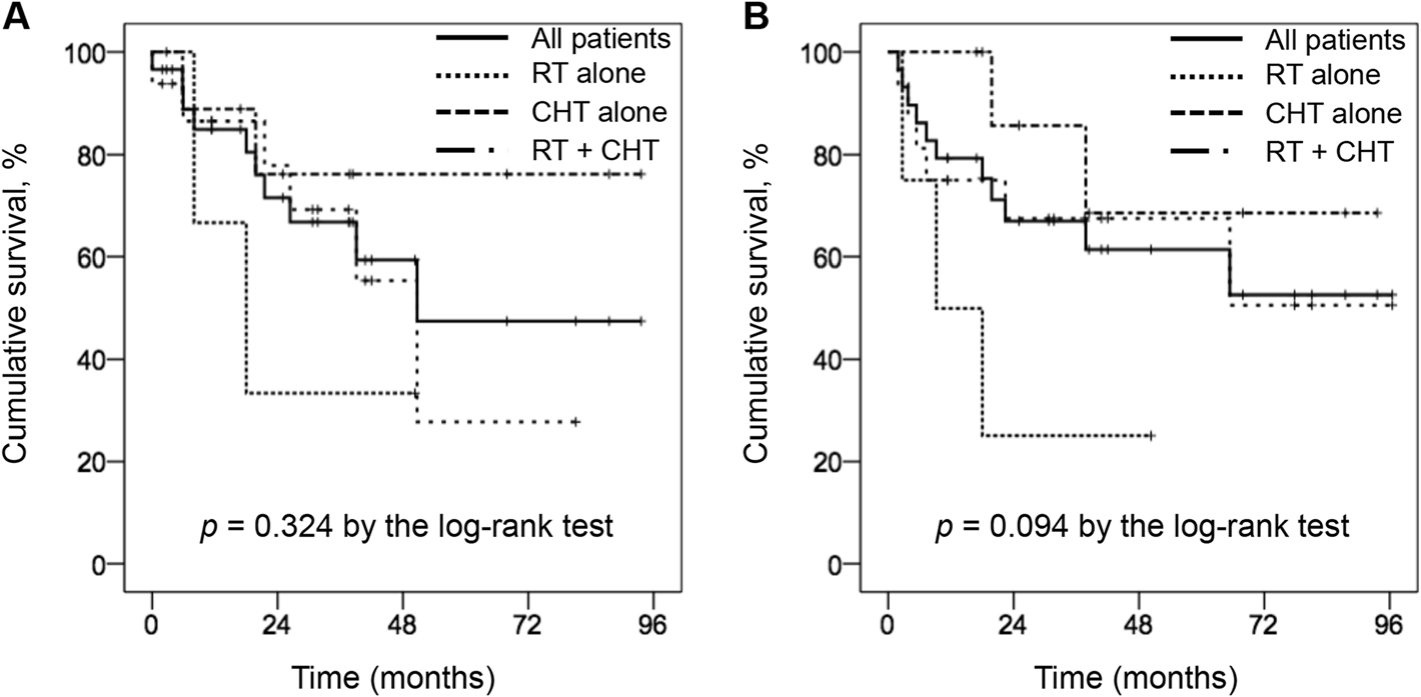
**Kaplan-Meier plot of (A) progression-free survival and (B) overall survival for all patients (n = 33), and patients treated with radiotherapy alone (n = 4), chemotherapy alone (n = 16), and chemoradiotherapy (n = 9).** RT, radiotherapy; CHT, chemotherapy.

The median follow-up was 40 months for the surviving patients. The median PFS for all patients was 51 months with the 5-year PFS rate being 43% (Figure 2A). The 5-year PFS rate was better in patients treated with chemoradiotherapy (76%) compared to patients treated with radiotherapy alone (33%) or chemotherapy alone (28%). However, this result was not statistically significant (*p* = 0.324). The median OS for all patients was 65 months, with the 5-year OS rate being 57% (Figure 2B). The 5-year OS rate was worse in patients treated with radiotherapy alone (25%) than patients treated with chemotherapy alone (68%) or chemoradiotherapy (69%). This result did not reach statistic significance (*p* = 0.094). During the follow-up period, 13 patients died, including two, five, four, one, and one from treatment-related causes after chemotherapy, renal failure, pneumonia, coronary artery occlusive disease, and cerebral hemorrhage after falling down, respectively.

The results of the univariate analyses for PFS and OS are shown in Table 5. In the univariate analyses, the use of chemotherapy was found to be a significant prognostic factor for PFS (3-year PFS, 71.5% versus 17.5%, *p* = 0.007) and OS (3-year OS, 74.1% versus 18.2%, *p* = 0.020). Moreover, VEGF elevation without gross lesions was found to be associated with a worse PFS (3-year PFS, 25.0% versus 66%, *p* = 0.035) and OS (3-year OS, 16.7% versus 74.1%, *p* = 0.008) compared with the other subtypes of POEMS syndrome.

**Table 5:** **Univariate prognostic factor analysis**

**Factor**	**No. of case**	**3-year PFS (%)**	***p*** **value** ^*^	**3-year OS (%)**	***p*** **value** ^*^
Age >50 years (vs. ≤50 years)	13/20	53.3/62.4	0.931	56.4/66.0	0.259
Radiotherapy (Y/N)	13/20	64.8/56.7	0.310	66.6/59.4	0.782
Chemotherapy (Y/N)	25/8	71.5/17.5	0.007	74.1/18.2	0.020
HD Chemotherapy with ABSCT (vs. SD chemotherapy)	15/10	80.8/58.3	0.291	78.8/66.7	0.253
IgG M-protein subtype (vs. other subtypes)	17/16	54.4/65.6	0.719	61.1/65.8	0.775
VEGF elevation without gross lesion (vs. other subtypes)	6/27	25.0/66.0	0.035	16.7/74.1	0.008
BM plasma cells more than 5% (vs. ≤5%)	13/20	61.4/59.5	0.771	60.6/62.5	0.821
The presence of abnormal clonal plasma cells in BM (Y/N)	5/28	60.0/60.8	0.249	80.0/60.4	0.552
Extravascular volume overload except peripheral edema (Y/N)	16/17	49.2/70.0	0.155	53.5/71.1	0.212
Number of POEMS features >7 (vs. ≤7)	14/19	46.4/67.7	0.220	45.9/74.2	0.127
Multiple bone lesions (vs. single bone lesion)	12/11	62.3/66.3	0.742	77.9/70.7	0.906

PFS, progression free survival; OS, overall survival; ABSCT, autologous peripheral blood stem cell transplantation; SD, standard dose; Ig, immunoglobulin; VEGF, vascular endothelial growth factor; BM, bone marrow.

^*^Log-rank test.

## Discussion

POEMS syndrome is a rare paraneoplastic syndrome resulting from underlying plasma cell proliferative diseases including OSM, CD, and VEGF elevation without gross lesion. Although a large case series of the disease was reported in the United States over a decade ago [6], the underlying pathogenesis and optimal treatment for POEMS syndrome remains unclear, partly due to the lack of randomized trials on the topic [7,16]. The results of the present study suggest that local radiotherapy could achieve durable clinical and hematologic responses in selected patients.

Osteosclerotic myeloma is characterized by osteosclerotic bone lesions caused by plasmacytoma or associated with POEMS syndrome. On the other hand, multiple myeloma is typically associated with osteolytic bone lesions [17], while bone lesions in cases of polyneuropathy are often osteosclerotic or mixed osteosclerotic and osteolytic [5,6]. Several studies have shown that OSM with a low burden of clonal plasma cells can be effectively treated with local radiotherapy [5,6,12,18-21]. Accordingly, in our study, we found that radiotherapy or radiotherapy plus chemotherapy produced a good clinical response of 89% and a hematologic response of 67%. Although the optimal dose of radiation for OSM has not been thoroughly investigated, moderate doses of over 40 Gy may be appropriate, as in the case of solitary plasmacytoma [12,22]. In the present study, only three patients received radiation doses less than 40 Gy, and among these three patients, one patient experienced disease progression. In addition, the two patients who did not respond to initial radiotherapy received 42 Gy and 50 Gy of radiotherapy. These doses were similar to those administered to the patients who responded to treatment, and were hence considered sufficient; therefore, the doses of radiation may not be responsible for the poor clinical responses, when radiation over 40 Gy was given.

Since disease multiplicity is associated with a higher risk of systemic involvement and a high burden of clonal plasma cells, it represents a very important factor to take into account for the decision of the treatment, especially, for patients with an underlying OSM. As a result, these patients are usually treated with chemotherapy, and the role of radiotherapy is thus uncertain in these patients. In the present study, six patients with multiple lesions underwent radiotherapy, and this was found to have effectively improved the POEMS associated symptoms, although the hematologic response to radiotherapy was relatively poor in these patients.

Furthermore, in the present study, among the six patients presenting with VEGF elevation without gross lesions, five patients were treated with systemic chemotherapy, whereas the other one patient received systemic corticosteroid therapy and intravenous immunoglobulin. Interestingly, the treatment responses of these patients were poorer than those of the patients with osteosclerotic myeloma (Table 4). Moreover, the PFS and OS rates were found to be significantly inferior in these patients compared to those in patients with other subtypes of underlying disease (Table 5). A French study reported that six out of 25 POEMS patients did not have bone lesion-like monoclonal gammopathy of unknown significance [23], and clinical improvement was observed in only one patient among four patients with available data in that series [5]. The reason for the poor clinical outcomes in these patients is unclear, and previous studies have reported only a small number of POEMS patients presenting with VEGF elevation without gross lesions; therefore, further investigations regarding these patients is needed.

In the present study, the administration of chemotherapy significantly improved PFS and OS. Previous studies have demonstrated that melphalan-based chemotherapy protocols were able to achieve response rates over 40% [4,6,15,24]. Moreover, high-dose chemotherapy with PBSCT has also produced encouraging results, with a 5-year PFS of 75% [11,13,25,26]. Therefore, the selection of patients indicated for chemotherapy and the choice of chemotherapy regimen based on the patients’ underlying clonal disease are important to improve outcomes.

The present study has some limitations including the retrospective nature and the relatively small sample size. Moreover, patients who did not undergo radiotherapy more frequently presented with adverse events such as ascites, pleural effusion, or pericardial effusion compared to patients treated with radiotherapy. Furthermore, among the 13 patients treated with radiotherapy, nine patients also underwent chemotherapy; and therefore, there is a possibility that the effects of radiotherapy may have been overestimated. However, despite these limitations, radiotherapy was found to produce good clinical responses in patients with a solitary lesion and clustered or limited multiple lesions, and radiotherapy may hence be effective as a definitive treatment in POEMS patients with localized OSM or as a palliative treatment to improve clinical symptoms in POEMS patients with clustered or limited multiple lesions.

## Conclusions

We here have shown that radiotherapy produced good clinical and hematologic responses, especially in patients with an underlying OSM. Additionally, radiotherapy and chemotherapy appear to both be effective treatments for underlying plasma cell proliferative diseases. Furthermore, we also found that patients with POEMS syndrome presenting with VEGF elevation without gross lesions tend to have a poor prognosis. Further large-scale studies are needed in the future to improve the understanding of the underlying disease pathogenesis and to elucidate the appropriate management of this disease based on patient characteristics.

## Abbreviations

OSM: Osteosclerotic myeloma
CD: Castleman’s disease
VEGF: Vascular endothelial growth factor
CBC: Complete blood cell count
WBBS: Whole body bone scan
CT: Computed tomography
Ig: Immunoglobulin
VGPR: Very good partial response
PR: Partial response
NR: No response
PFS: Progression-free survival
OS: Overall survival

## References

[1] Bardwick PA, Zvaifler NJ, Gill GN, Newman D, Greenway GD, Resnick DL (1980). Plasma cell dyscrasia with polyneuropathy, organomegaly, endocrinopathy, M protein, and skin changes: the POEMS syndrome. Report on two cases and a review of the literature. Med (Baltimore).

[2] Dispenzieri A (2007). POEMS syndrome. Blood Rev.

[3] Dispenzieri A (2014). POEMS syndrome: 2014 update on diagnosis, risk-stratification, and management. Am J Hematol.

[4] Nakanishi T, Sobue I, Toyokura Y, Nishitani H, Kuroiwa Y, Satoyoshi E, Tsubaki T, Igata A, Ozaki Y (1984). The crow-fukase syndrome: a study of 102 cases in Japan. Neurology.

[5] Soubrier MJ, Dubost JJ, Sauvezie BJ, French Study Group on POEMS Syndrome (1994). POEMS syndrome: a study of 25 cases and a review of the literature. Am J Med.

[6] Dispenzieri A, Kyle RA, Lacy MQ, Rajkumar SV, Therneau TM, Larson DR, Greipp PR, Witzig TE, Basu R, Suarez GA, Fonseca R, Lust JA, Gertz MA (2003). POEMS syndrome: definitions and long-term outcome. Blood.

[7] Dispenzieri A (2011). POEMS syndrome: 2011 update on diagnosis, risk-stratification, and management. Am J Hematol.

[8] Li J, Tian Z, Zheng HY, Zhang W, Duan MH, Liu YT, Cao XX, Zhou DB (2013). Pulmonary hypertension in POEMS syndrome. Haematologica.

[9] Soubrier M, Dubost JJ, Serre AF, Ristori JM, Sauvezie B, Cathebras P, Piette JC, Chapman A, Authier FJ, Gherardi RK (1997). Growth factors in POEMS syndrome: evidence for a marked increase in circulating vascular endothelial growth factor. Arthritis Rheum.

[10] Dispenzieri A (2012). How I treat POEMS syndrome. Blood.

[11] D'Souza A, Lacy M, Gertz M, Kumar S, Buadi F, Hayman S, Dingli D, Zeldenrust S, Kyle R, Ansell S, Inwards D, Johnston P, Micallef I, Porrata L, Litzow M, Gastineau D, Hogan W, Dispenzieri A (2012). Long-term outcomes after autologous stem cell transplantation for patients with POEMS syndrome (osteosclerotic myeloma): a single-center experience. Blood.

[12] Humeniuk MS, Gertz MA, Lacy MQ, Kyle RA, Witzig TE, Kumar SK, Kapoor P, Lust JA, Hayman SR, Buadi FK, Rajkumar SV, Zeldenrust SR, Russell SJ, Dingli D, Lin Y, Leung N, Dispenzieri A (2013). Outcomes of patients with POEMS syndrome treated initially with radiation. Blood.

[13] Jaccard A, Royer B, Bordessoule D, Brouet JC, Fermand JP (2002). High-dose therapy and autologous blood stem cell transplantation in POEMS syndrome. Blood.

[14] Plata Bello J, Garcia-Marin V (2013). POEMS syndrome (polyneuropathy, organomegaly, endocrinopathy, multiple myeloma and skin changes) with cranial vault plasmocytoma and the role of surgery in its management: a case report. J Med Case Rep.

[15] Li J, Zhou DB, Huang Z, Jiao L, Duan MH, Zhang W, Zhao YQ, Shen T (2011). Clinical characteristics and long-term outcome of patients with POEMS syndrome in China. Ann Hematol.

[16] Dispenzieri A (2012). POEMS syndrome: update on diagnosis, risk-stratification, and management. Am J Hematol.

[17] Evison G, Evans KT (1967). Bone sclerosis in multiple myeloma. Br J Radiol.

[18] Morley JB, Schwieger AC (1967). The relation between chronic polyneuropathy and osteosclerotic myeloma. J Neurol Neurosurg Psychiatry.

[19] Iwashita H, Ohnishi A, Asada M, Kanazawa Y, Kuroiwa Y (1977). Polyneuropathy, skin hyperpigmentation, edema, and hypertrichosis in localized osteosclerotic myeloma. Neurology.

[20] Davis LE, Drachman DB (1972). Myeloma neuropathy. Successful treatment of two patients and review of cases. Arch Neurol.

[21] Reitan JB, Pape E, Fossa SD, Julsrud OJ, Slettnes ON, Solheim OP (1980). Osteosclerotic myeloma with polyneuropathy. Acta Med Scand.

[22] Suh YG, Suh CO, Kim JS, Kim SJ, Pyun HO, Cho J (2012). Radiotherapy for solitary plasmacytoma of bone and soft tissue: outcomes and prognostic factors. Ann Hematol.

[23] International Myeloma Working G (2003). Criteria for the classification of monoclonal gammopathies, multiple myeloma and related disorders: a report of the international myeloma working group. Br J Haematol.

[24] Stewart PM, McIntyre MA, Edwards CR (1989). The endocrinopathy of POEMS syndrome. Scott Med J.

[25] Ganti AK, Pipinos I, Culcea E, Armitage JO, Tarantolo S (2005). Successful hematopoietic stem-cell transplantation in multicentric Castleman disease complicated by POEMS syndrome. Am J Hematol.

[26] Wiesmann A, Weissert R, Kanz L, Einsele H (2002). Long-term follow-up on a patient with incomplete POEMS syndrome undergoing high-dose therapy and autologous blood stem cell transplantation. Blood.

